# Binding of IscU and TusA to different but competing sites of IscS influences the activity of IscS and directs sulfur to the respective biomolecular synthesis pathway

**DOI:** 10.1128/spectrum.00949-24

**Published:** 2024-07-09

**Authors:** Paolo Olivieri, Moritz Klabes, Jason C. Crack, Angelika Lehmann, Sophie P. Bennett, Nick E. Le Brun, Silke Leimkühler

**Affiliations:** 1 Department of Molecular Enzymology, Institute of Biochemistry and Biology, University of Potsdam, Potsdam, Germany; 2 Centre for Molecular and Structural Biochemistry, School of Chemistry, University of East Anglia, Norwich, United Kingdom; Forschungszentrum Jülich GmbH, Juelich, Germany

**Keywords:** IscS, Fe-S cluster, iron, sulfur, cysteine desulfurase

## Abstract

All sulfur transfer pathways generally have in common an l-cysteine desulfurase as the initial sulfur-mobilizing enzyme, which serves as a sulfur donor for the biosynthesis of numerous sulfur-containing biomolecules in the cell. In *Escherichia coli*, the housekeeping l-cysteine desulfurase IscS functions as a hub for sulfur transfer through interactions with several partner proteins, which bind at different sites on IscS. So far, the interaction sites of IscU, Fdx, CyaY, and IscX involved in iron sulfur (Fe-S) cluster assembly, TusA, required for molybdenum cofactor biosynthesis and mnm^5^s^2^U34 transfer RNA (tRNA) modifications, and ThiI, involved in both the biosynthesis of thiamine and s^4^U8 tRNA modifications, have been mapped. Previous studies have suggested that IscS partner proteins bind only one at a time, with the exception of Fe-S cluster assembly, which involves the formation of a ternary complex involving IscS, IscU, and one of CyaY, Fdx, or IscX. Here, we show that the affinity of TusA for IscS is similar to but lower than that of IscU and that these proteins compete for binding to IscS. We show that heterocomplexes involving the IscS dimer and single IscU and TusA molecules are readily formed and that binding of both TusA and IscU to IscS affects its l-cysteine desulfurase activity. A model is proposed in which the delivery of sulfur to different sulfur-requiring pathways is controlled by sulfur acceptor protein levels, IscS-binding affinities, and acceptor protein-modulated IscS desulfurase activity.

**IMPORTANCE:**

Iron-sulfur clusters are evolutionarily ancient prosthetic groups. The housekeeping l-cysteine desulfurase IscS functions as a central core for sulfur transfer through interactions with several partner proteins, which bind at different sites on each IscS monomer with different affinities and partially overlapping binding sites. We show that heterocomplexes involving the IscS dimer and single IscU and TusA molecules at each site of the dimer are formed, thereby influencing the activity of IscS.

## INTRODUCTION

Sulfur is an essential element for all living organisms (1). In bacteria, sulfur is present in a variety of cofactors and biomolecules such as thiamin, iron-sulfur (Fe-S) clusters, biotin, lipoic acid, molybdopterin (MPT), and thionucleosides in transfer RNA (tRNA) (1). In the last few years, several studies of cellular sulfur transfer pathways concluded that the major enzymes involved in the mobilization of sulfur are l-cysteine desulfurases (2). These enzymes catalyze the formation of a persulfide group (R^1^–S–SH) on specific conserved cysteine residues, which can form a hetero-disulfide bond (R^1^–S–S^0^–S–R^2^) with a target molecule as intermediate (1, 3). This provides a route for cellular sulfur transfer from a donor protein to an acceptor protein without increasing the soluble sulfur concentrations to toxic levels. Especially in the last few years, it has been discovered that the assembly of Fe-S clusters, the biosynthesis of the molybdenum cofactor (Moco), and the synthesis of thio-modified tRNAs share the same protein complex for essential sulfur mobilization (4).

*Escherichia coli* contains the house-keeping ISC system (for iron-sulfur cluster assembly) that is involved in the assembly of Fe-S clusters under optimal growth conditions (5). A second system, called SUF (for sulfur mobilization), functions to assemble Fe-S clusters under conditions of iron limitation or oxidative stress (2). The *isc*-operon is composed of the genes *iscRSUA-hscBA-fdx-iscX,* and its transcription is controlled by IscR, a [2Fe-2S] cluster-binding transcriptional repressor (5). The other genes in the operon encode for the l-cysteine desulfurase IscS, the scaffold protein IscU, a [2Fe-2S] cluster-containing ferredoxin Fdx, the A-type carrier protein IscA, the molecular chaperones HscA and HscB, and the likely regulatory protein IscX (6). The scaffold protein IscU is central to Fe-S cluster biosynthesis, being responsible for the assembly of [2Fe-2S] and [4Fe-4S] clusters on client proteins (7). IscS catalyzes the pyridoxal phosphate (PLP)-dependent breakdown of l-cysteine to l-alanine, releasing inorganic sulfur for Fe-S clusters (8, 9). Fdx donates electrons for cluster assembly, and IscA is one of the carrier proteins that is involved in the transfer of assembled Fe-S clusters to target proteins (10, 11). The exact function of IscX is unknown, but it has been proposed to act in conjunction with CyaY to allosterically modulate the assembly of Fe-S clusters (12–14). CyaY is the only protein with a role in Fe-S cluster assembly that is not encoded by the *isc*-operon (15).

The human homolog of CyaY is frataxin (FXN), to which a potential role in assembly of Fe-S clusters and regulation of iron homeostasis has been assigned (16). In *E. coli*, CyaY was shown to regulate the activity of IscS by slowing down the rate of Fe-S cluster formation *in vitro*, while *in vivo* studies showed that, in the cellular environment, it serves to promote Fe-S assembly (17). In contrast, FXN was shown to activate Fe-S cluster assembly both *in vitro* and in mitochondria of eukaryotes (18). This opposing *in vitro* effect, however, activation or inhibition, was shown to be dependent on the nature of the cysteine desulfurase (18).

In addition to IscU, Fdx, CyaY, and IscX, several other partner proteins were identified as interaction partners of IscS, including TusA for Moco biosynthesis and the mnm^5^s^2^U34 thiomodification in tRNAs, and ThiI for both thiamine biosynthesis and s^4^U8 tRNA modifications (19). Overall, there is a complex protein-protein interaction network around IscS, being the master enzyme in the initial mobilization of sulfur from l-cysteine and its transfer, in form of a persulfide, onto specific sulfur acceptor proteins (4). So far, the interaction sites of IscU, ThiI, TusA, IscX, Fdx, and CyaY have been mapped on IscS (19). Previous studies predicted that the sulfur acceptor proteins bind to IscS only one at a time (11). The exception seems to be the Fe-S cluster assembly pathway that involves the formation of a ternary complex on IscS, consisting of IscU and one of CyaY, Fdx, or IscX (12, 14). However, it remains unclear how the preference for the specific interaction partner is regulated and, thus, how sulfur transfer is directed into specific biomolecular pathways. Models have been proposed in which the concentration of the sulfur acceptor protein is the determining factor for preferred binding to IscS under particular growth conditions (19–21).

For Moco biosynthesis, two sulfur atoms are inserted sequentially into the first stable intermediate, cyclic pyranopterin monophosphate (cPMP) (22). Previous results identified the small sulfur transfer protein TusA to be involved in this step of Moco biosynthesis (20). Here, the molybdopterin synthase, consisting of two MoaD and MoaE subunits, performs the direct insertion of two sulfur atoms into cPMP to form MPT (23). The immediate sulfur donor in this reaction is the thiocarboxylate group at the C-terminal glycine of MoaD, present in the MPT synthase complex (23, 24). The formation of the thiocarboxylate group on MoaD directly requires MoeB (25, 26). In the course of the regeneration reaction, MoeB and MoaD form a tetrameric (MoaD-MoeB)_2_ complex in which an acyl-adenylate group is formed at the C-terminal glycine of MoaD under ATP consumption (25, 26). In its activated form, sulfur is directly transferred from TusA to MoaD-AMP in the (MoaD-MoeB)_2_ complex, and thiocarboxylated MoaD-SH is formed.

Besides its role in Moco biosynthesis, TusA was initially described as being involved in the formation of mnm^5^s^2^U34-modified tRNA for Lys, Gln, and Glu (27). Thiomodifications of tRNAs are important for proper function of pro- and eukaryotic organisms (28, 29). To date, four different thionucleosides have been identified at different positions in several prokaryotic tRNAs: 4-thiouridine at position 8 (s^4^U8), 2-thiocytidine at position 32 (s^2^C32), 5-methylaminomethyl-2-thiouridine at position 34 (mnm^5^s^2^U34), and 2-methylthio-N6 isopentenyladenosine at position 37 (ms^2^i^6^A37) (28). For the formation of mnm^5^s^2^U in tRNA for Lys, Gln, and Glu in *E. coli*, a sulfur-relay system was identified, including initial sulfur mobilization by the l-cysteine desulfurase IscS and the proteins TusA, TusBCD, TusE, and MnmA (27). The *tusB*, *tusC,* and *tusD* genes are located at a single operon, and the encoded proteins form a dimeric heterotrimer (TusBCD)_2_ complex. Efficient 2-thiouridine formation can be achieved *in vitro* by incubating purified tRNA with IscS, TusA, (TusBCD)_2_, TusE, and MnmA (27). TusA thereby directly interacts with IscS, stimulates its desulfurase activity, and directs the sulfur flow to 2-thiouridine formation.

Overall, TusA has a dual role in the cell, delivering the sulfur required for both the thiomodification of thionucleosides in tRNA and for Moco biosynthesis (4). Deletion of *tusA* was found to have a pleiotropic effect on several cellular pathways in *E. coli*, not only including tRNA thiolation and Moco biosynthesis but also on the enhanced susceptibility of viral infection inhibition by programmed ribosomal frameshifting (21). These pleiotropic effects were suggested to be caused by changes in the Fe-S cluster concentration in the cell, suggesting a link between Fe-S cluster availability and tRNA thiolation/Moco biosynthesis (20). Studies showed that elevated levels of TusA in *E. coli* decreased Fe-S cluster availability. A consequence of this is that Fe-S cluster-containing proteins such as MoaA exhibited lower activity, which directly resulted in a decreased activity of molybdoenzymes. On the other hand, overexpression of IscU (scaffold for Fe-S cluster assembly) also reduced the level of active molybdoenzymes in *E. coli* (20). This observation was explained on the basis of an increase in complex formation between IscU and IscS, thereby limiting IscS availability for interaction with other proteins such as TusA. Thus, TusA and IscU appear to play a role in regulating the availability of IscS for the different sulfur transfer pathways. In the absence of TusA, the availability of IscS for Fe-S cluster assembly may increase (20), while sulfur transfer from IscS to other biosynthetic pathways, such as Moco or thiolated tRNA, would be reduced.

Overall, it is clear that the pathways for sulfur transfer to sulfur-containing biomolecules, such as Moco biosynthesis, Fe-S cluster assembly, and thiolation of tRNAs, are tightly connected, and it is likely that they are regulated at a cellular level, by, for example, the availability of their acceptor proteins and possibly other factors. In this study, we have further investigated TusA binding to IscS and explored the effects of mixtures of IscS with IscU and TusA on complex formation, and desulfurase and Fe-S cluster assembly activities. A model of the control of sulfur transfer in the cell is discussed.

## RESULTS

### The effect of TusA and IscU on IscS l-cysteine desulfurase activity

**Fig 1:**
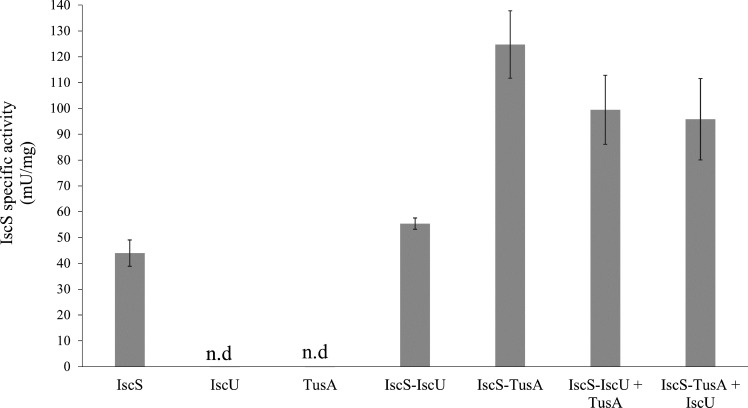
TusA and IscU alter the l-cysteine desulfurase activity of preformed IscS complexes. The l-cysteine desulfurase activity of purified IscS or preformed complexes of IscS/IscU and IscS/TusA was measured by the methylene blue colorimetric assay, and the effect of the addition of either TusA or IscU was tested. For precomplex formation, IscS was incubated with IscU or TusA, respectively, in a 1:3 ratio for 20 min at 30°C. The complexes were purified using a Superdex 200 pg column. To the purified, preformed IscS/IscU and IscS/TusA complexes, either TusA or IscU was added in a 1:3 ratio, followed by incubation for 10 min at 30°C in the presence of 1 mM DTT and 1 mM l-cysteine. The reaction was stopped, and the produced sulfide was quantified using an Na_2_S (0–200 µM) standard curve. n.d, no activity detected.

It was shown previously that TusA enhances the activity of IscS about threefold (20). To determine how this enhancement is influenced by the presence of IscU, l-cysteine desulfurase activity assays, with quantification through the formation of methylene blue (7), were performed. In addition to *in vitro* measurements of IscS alone and IscS in complex with TusA or IscU, the effect of adding either TusA or IscU to preformed complexes of IscS with, respectively, IscU or TusA, was investigated (Fig. 1).

As expected, the addition of TusA to IscS resulted in an increase in the IscS activity by a factor of about 3. The addition of IscU alone to IscS resulted only in a slight increase in desulfurase activity. In contrast, when TusA was added to the preformed IscS-IscU complex, the activity was increased but not to the level of the IscS-TusA complex alone. When IscU was added to the preformed IscS-TusA complex, the activity was decreased but again not to the level of the IscS-IscU complex alone. The data indicate that TusA and IscU influence the preformed IscS-IscU or IscS-TusA complexes, most likely through direct competition.

### Analytical gel filtration studies of complex formation involving IscS

The interaction sites of TusA, CyaY, and IscU on IscS were mapped previously and excluded a simultaneous interaction of all four proteins, as confirmed by co-affinity purification experiments (19). A ternary complex of IscS, IscU, and CyaY is known to form, but the influence of TusA on this complex, or on the IscS-IscU complex, has not yet been investigated by gel filtration studies. In general, previous studies of protein complex formation have employed tagged versions of IscS and the other proteins. We found that a His-tag at the N-terminus of IscS may not be innocent since His_10_-tagged IscS had a negative effect on the growth of an *iscS* deletion strain after complementation with the plasmid (Fig. S1). We note that overexpression of *iscS* by itself deregulates sulfur transfer from IscS to its acceptors (20) and thus does not fully complement the isc mutant (Fig. S1). The negative effect of the His_10_-tag might be based on an interference with the binding site of interaction partners since it is more pronounced than the effect of overexpressing untagged *iscS* alone (Fig. S1). Therefore, to avoid influences of affinity tags on protein-protein interactions, we used untagged versions of TusA and IscS for interaction studies. To gain deeper insight into protein complex formation on IscS, with multiple simultaneous binding partners, analytical gel filtration experiments were conducted using preformed protein complexes of either IscS-IscU or IscS-TusA, to which the second putative interaction partner, TusA or IscU, was added and analyzed via a second gel filtration run.

**Fig 2:**
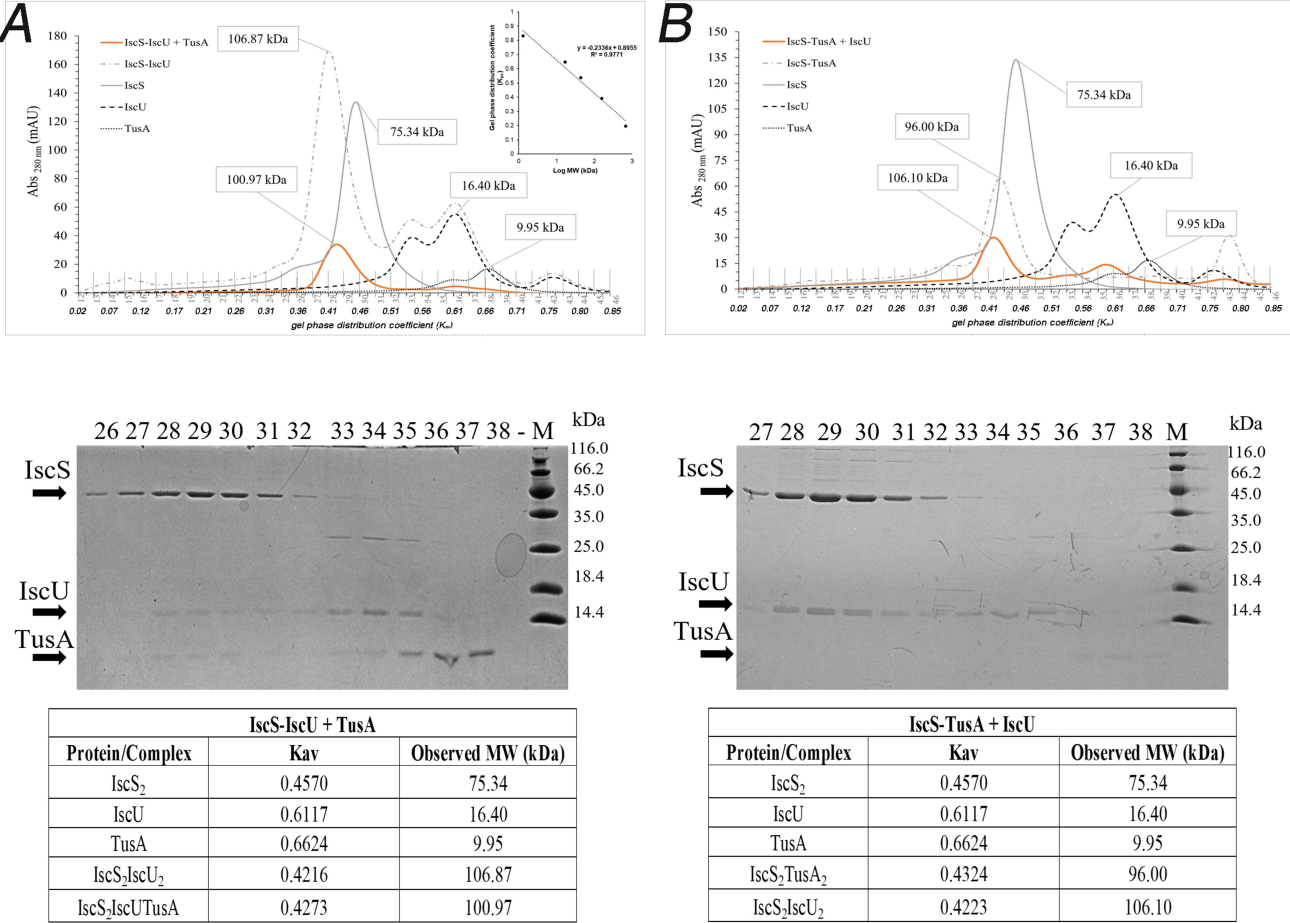
Analysis of putative IscS-IscU-TusA heterocomplex formation by usage of analytical size exclusion chromatography and SDS-PAGE. The preformed complexes IscS-IscU (**A**) and IscS-TusA (**B**) were first isolated by analytical size exclusion chromatography (Superdex 200) after incubation of IscS with IscU (**A**) or with TusA (**B**) in a 1:3 ratio for 20 min at 30°C. The fractions containing the respective complexes were combined and concentrated. Using the same protocol, the complex IscS-IscU was incubated with TusA (**A**), and the IscS-TusA complex was incubated with IscU (**B**) in a 1:3 ratio. The inset shows the calibration of the gel-phase distribution coefficient (*K*_av_) to determine the molecular weight using standard proteins. After elution (orange chromatograms), the indicated fractions were separated using 17% SDS-PAGE, using an unstained protein weight ladder as marker (Thermo Scientific; 14.4–116.0 kDa). The band at 35 kDa is the LacI protein that is often copurified with pET-expressed proteins. That was confirmed by mass spectrometry. The tables (**A and B**) show *K*_av_ and the calculated molecular masses (from a standard curve) of the single proteins and the protein complexes for each chromatography run.

The preformed IscS_2_-IscU_2_ complex eluted from the column at a *K*_av_ of 0.42. When TusA was added to the IscS_2_-IscU_2_ complex, elution occurred at *K*_av_ of 0.43, with all three proteins being present in the major complex fractions, implying an IscS_2_-IscU-TusA heterocomplex (Fig. 2A).

The preformed IscS_2_-TusA_2_ complex eluted at *K*_av_ of 0.43, with both proteins present in the fractions of interest (Fig. 2B). When IscU was added to the IscS_2_-TusA_2_ precomplex, the complex eluted at a *K*_av_ of 0.42 with a similar elution volume to that of the IscS_2_-IscU_2_ complex, with both IscS and IscU present in the corresponding fractions, and no band due to TusA observed, as shown by Coomassie-stained SDS-gel (Fig. 2B). This might imply that IscU displaced TusA from the IscS complex, as shown previously (19), and a IscS_2_-IscU_2_ complex was formed. However, as noted above, when TusA was added to the preformed IscS_2_-IscU_2_ complex, the data suggested the possible formation of a ternary IscS-IscU-TusA complex, and so further investigation was necessary.

### Native mass spectrometry of IscS complexes

**Fig 3:**
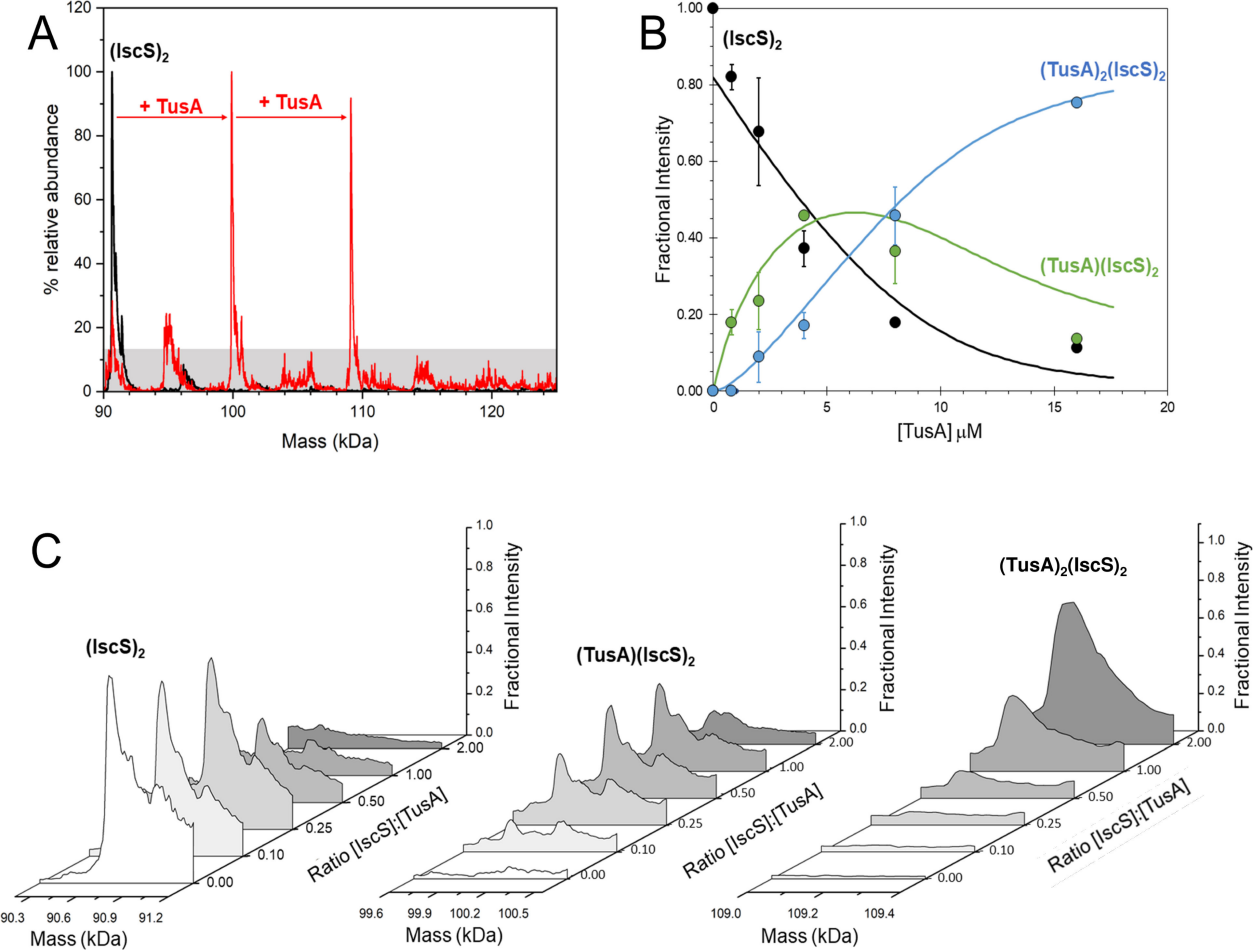
ESI-MS investigation of complex formation between IscS and TusA. (**A**) Deconvoluted mass spectrum of IscS over the mass range 90–125 kDa, showing the presence of the IscS dimer (black spectrum). Addition of TusA at a 2:1 excess gave rise to TusA-IscS complexes in which the IscS dimer is bound by one or two TusA protein molecules (red spectrum). (**B**) Deconvoluted mass spectral intensity at increasing concentrations of TusA (and, hence, ratios of TusA to IscS) corresponding to the various protein complexes, as indicated. Solid lines show fits of the data to a sequential binding model for one to two TusA per IscS dimer. (**C**) Spectral regions corresponding to the various protein complexes, as indicated, at increasing TusA to IscS ratios. IscS (4 µM) was in 250 mM ammonium acetate, pH 8. Note that abundances are reported relative to the most abundant species, which is arbitrarily set to 100%.

To complement analytical gel filtration experiments, electrospray ionization mass spectrometry (ESI-MS) under non-denaturing conditions (native MS) was applied. This approach, if optimized, can provide direct information on all protein complexes present in the solution. The *m/z* spectrum of dimeric IscS displayed well-resolved charge states (Fig. S2), as previously observed (6, 12, 14). The deconvoluted spectrum of IscS revealed a minor peak at 90,646 Da corresponding to (IscS)_2_ with its PLP cofactors (predicted mass: 90,642 Da) together with a major peak at 90,662 Da, likely due to a sodium adduct of the IscS complex (predicted mass: 90,663 Da) (Fig. 3A). Initial experiments revealed that the addition of IscU and/or TusA to IscS resulted in the formation of new charge states (Fig. S2 shows data for addition of TusA to IscS) that could be deconvoluted to neutral mass to reveal the presence of IscS-TusA-IscU complexes, consistent with gel filtration observations. To investigate these complexes further, dimeric IscS and associated complexes were titrated with increasing amounts of TusA and/or IscU.

### IscS has two binding sites for TusA

The addition of TusA (Fig. S3) to dimeric IscS, to a final 4:1 molar ratio, resulted in significant changes in the *m/z* spectrum (Fig. S2). A new pattern of charge states superimposed over those of IscS was observed, consistent with the presence of IscS-TusA complexes. The deconvoluted spectrum (Fig. 3, spanning 90–140 kDa) revealed the presence of two new species, corresponding to dimeric IscS in complex with up to two TusA molecules at intervals of 9,230 Da (predicted mass 9,226 Da). When increasing concentrations of TusA were added to IscS, the sequential formation of complexes containing 1 and 2 TusA molecules (Fig. 3A) was observed. The complex of dimeric IscS with a single TusA molecule, (TusA)(IscS)_2_, formed readily at low levels of TusA ([TusA]:[IscS] ≈ 0.1) and maximized at [TusA]:[IscS] ≈ 0.5. The (TusA)_2_(IscS)_2_ complex was detectable at a [TusA]:[IscS] ratio of ~0.25 and reached a maximum abundance by [TusA]:[IscS] ratio ≈ 2. The data were analyzed according to a sequential binding model, which indicated that the binding of TusA molecules to form (TusA)(IscS)_2_ or (TusA)_2_(IscS)_2_ occurred with a similar affinity, *K*_*d*_ ≈ 8 µM. As the binding behavior was well described by a single average dissociation constant, and there was no evidence for cooperativity, we conclude that the IscS dimer contains two equivalent, but independent, binding sites for TusA, consistent with one TusA site on each IscS monomer.

### The surface arginine patch of IscS is important for interaction with TusA

Three arginine residues (Arg220, Arg223, and Arg225) located on the surface of IscS have been shown to participate in protein-protein interactions. Structural studies of the interaction between *E. coli* IscS and TusA resulted in two crystal forms with identical heterotetramers consisting of an IscS dimer and two TusA molecules (19). The structure revealed a salt bridge between the proteins involving Arg220 of IscS (and Glu21 of TusA) (19). Replacement of the Arg220, Arg223, and Arg225 residues with glutamates abolished the interaction of IscS with the accessory proteins IscX and CyaY, but not with IscU, whose binding site does not overlap with those of IscX/CyaY (12). To determine the extent to which the binding of TusA to IscS is affected by these substitutions, the R220/223/225E triple variant of IscS (^EEE^IscS) was titrated, as described above, with increasing amounts of TusA. Initial native MS observations confirmed that the triple variant of IscS, like wild-type IscS, is dimeric with a full complement of PLP cofactor and a mass of 90,874 Da (predicted mass 90,878 Da). When increasing concentrations of TusA were added to ^EEE^IscS, the sequential formation of complexes containing 1 and 2 TusA molecules per IscS was observed (Fig. S4). The complex of dimeric ^EEE^IscS with a single TusA molecule, (TusA)(^EEE^IscS)_2_, formed readily at low levels of TusA ([TusA]:[ ^EEE^IscS] ≥0.1) and maximized at [TusA]:[ ^EEE^IscS] ≥1.3. The (TusA)_2_(IscS)_2_ complex was detectable at [TusA]:[ ^EEE^IscS] ratios ≥0.5 but never reached a maximum during the titration. The data were again analyzed according to a sequential binding model, revealing that the binding of TusA to form (TusA)(^EEE^IscS)_2_ or (TusA)_2_(^EEE^IscS)_2_ occurred independently with a similar affinity, *K*_*d*_ = ~50 µM. This is significantly higher than for the wild-type protein, indicating that the interaction of ^EEE^IscS with TusA is disrupted but not abolished entirely (Fig. S4).

### Cys variants of TusA bind to IscS with affinities similar to wild-type TusA

TusA has two cysteine residues, Cys19 and Cys56, both of which are important for function. Cys19 is essential for persulfide transfer from IscS and delivery to client proteins, while Cys56 is involved in intermolecular interactions between TusA and client proteins (27). The crystal structure of the IscS-TusA complex revealed the interaction of TusA almost entirely with the large domain of one IscS subunit within the dimer, with the exception being the tip of the loop containing the essential Cys328 of IscS, which comes from the other subunit. This persulfide-carrying Cys328 is juxtaposed against the acceptor cysteine (Cys19) of TusA with only 4 Å separating their sulfur atoms, a distance short enough to enable sulfur transfer to occur. Cys56 of TusA does not make any direct contacts to IscS residues (19).

To determine if the binding of TusA to IscS is affected in the absence of these residues, C19S and C56A variants were generated, and solutions of IscS were titrated with the TusA variants, as described above. In general, the TusA variants behaved like wild-type TusA with (^C→S/A^TusA)(IscS)_2_ complexes forming readily at low levels of TusA ([TusA]:[IscS] ≥0.1) and maximizing around [^C→S/A^TusA]:[IscS] ≈ 1.0. The (^C→S/A^TusA)_2_(IscS)_2_ complexes were detectable at a [TusA]:[IscS] ratio of ~0.25 and reached a maximum abundance when [TusA]:[IscS] ratios were ≥2. Analysis using the sequential binding model resulted in satisfactory fits to the data, revealing affinities comparable to those of wild-type TusA, with average *K*_*d*_ values for both of ~8 µM (Fig. S5). These observations indicate that neither Cys19 nor Cys56 plays important roles in the interaction of TusA with IscS, consistent with previous studies that showed that residues located elsewhere are responsible for maintaining protein-protein interactions in the TusA-IscS complex (19).

### IscS preferentially binds IscU over TusA

Structures of the IscU-IscS and TusA-IscS complexes show that IscU and TusA recognize distinct, but adjacent, binding sites that provide access to the active site Cys328 of IscS (19), raising the possibility that higher-order mixed complexes of IscS with TusA and IscU may be possible. We note that the location of TusA on IscS is largely comparable to other accessory proteins (e.g., CyaY and IscX) but, unusually, that the TusA and IscU binding sites also partially overlap, suggesting that TusA may compete with IscU for IscS.

**Fig 4:**
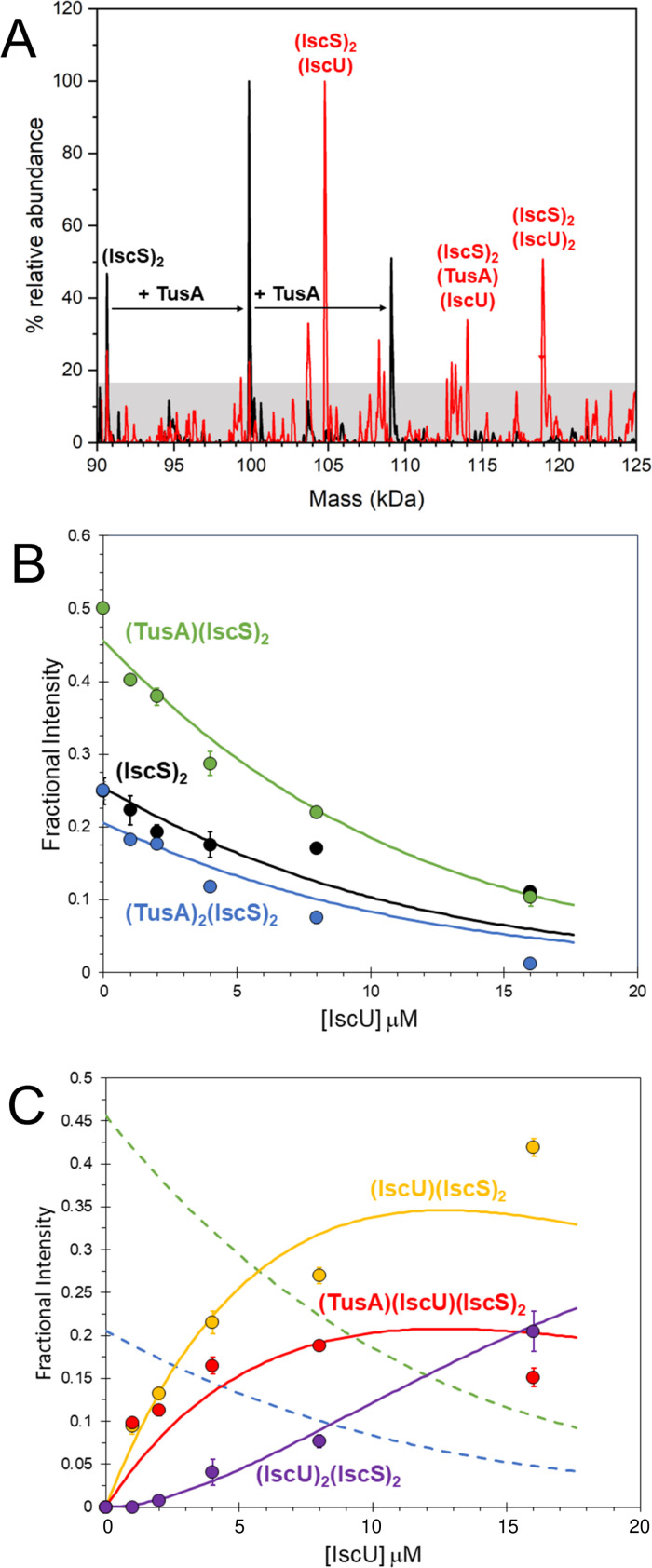
ESI-MS investigation of complex formation between IscS, TusA, and IscU. (**A**) Deconvoluted mass spectrum of IscS over the mass range 90–125 kDa, showing the presence of the IscS dimer and TusA complexes (black spectrum) resulting from the addition of TusA at a 2:1 excess. Subsequent addition of IscU gave rise to additional complexes, including an (IscS)_2_(TusA)(IscU) species (red spectrum). (**B**) Plots of relative intensity of the (IscS)_2_ and TusA-IscS complexes during titration with IscU, as indicated, as a function of IscU concentration. (**C**) As in (**B**) but for IscU-bound IscS complexes. Solid lines show fits of the data to a competition binding model for one to two TusA or IscU molecules per IscS dimer. IscS (8 µM) was in 250 mM ammonium acetate, pH 8.

Initially, IscS (8 µM IscS, 4 µM dimeric IscS) was pretreated with a molar equivalent of TusA (8 µM) to generate TusA-IscS complexes. The predominant species in the native MS spectrum was (TusA)(IscS)_2_, followed by (IscS)_2_ then (TusA)_2_(IscS)_2_. To investigate the effect of IscU on these complexes, the sample was titrated with increasing amounts of IscU (Fig. 4). This resulted in the formation of (TusA)(IscU)(IscS)_2_, (IscU)(IscS)_2_, and (IscU)_2_(IscS)_2_ and concomitant decline of (TusA)(IscS)_2_, (TusA)_2_(IscS)_2_, and (IscS)_2_ complexes. The mixed heterocomplex (TusA)(IscU)(IscS)_2_ formed readily at low levels of IscU ([IscU]:[IscS] ≈ 0.2) and maximized at [IscU]:[IscS] ≥1.

Unlike for other accessory proteins, such as IscX and CyaY, the addition of IscU to TusA-IscS complexes did not lead to formation of a (TusA)_2_(IscU)_2_(IscS)_2_ complex, an observation consistent with distinct, but overlapping, TusA/IscU-binding sites. Hence, the data were analyzed according to a simple competition binding model (Fig. 4). The resulting fit of the data indicated that complex formation is governed by the higher affinity of IscU [*K*_*d*_ = ~3 µM, as previously determined (30)] over TusA (*K*_*d*_ = ~8 µM) for the adjacent, but overlapping, binding site.

Reciprocal experiments were performed in which preformed IscU-IscS complexes were titrated with TusA (Fig. S6). The formation of (TusA)(IscU)(IscS)_2_, (TusA)(IscS)_2_, and (TusA)_2_(IscS)_2_ complexes was observed, along with concomitant decline of (IscU)(IscS)_2_ and (IscS)_2_ complexes. The mixed heterocomplex (TusA)(IscU)(IscS)_2_ was also observed. However, the titration data (Fig. S6B) indicate that, compared to the titration of the preformed TusA-IscS complexes with IscU, higher concentrations of TusA were required in order to displace IscU, consistent with the preferred binding of IscS to IscU over TusA. Taken together, the native MS and gel filtration experiments reported here are consistent with the idea that TusA and IscU compete for discrete, but overlapping, binding sites on IscS and that each monomer of the IscS dimer can independently accommodate either IscU or TusA but not both.

### Effect of TusA on *in vitro* Fe-S cluster formation

**Fig 5:**
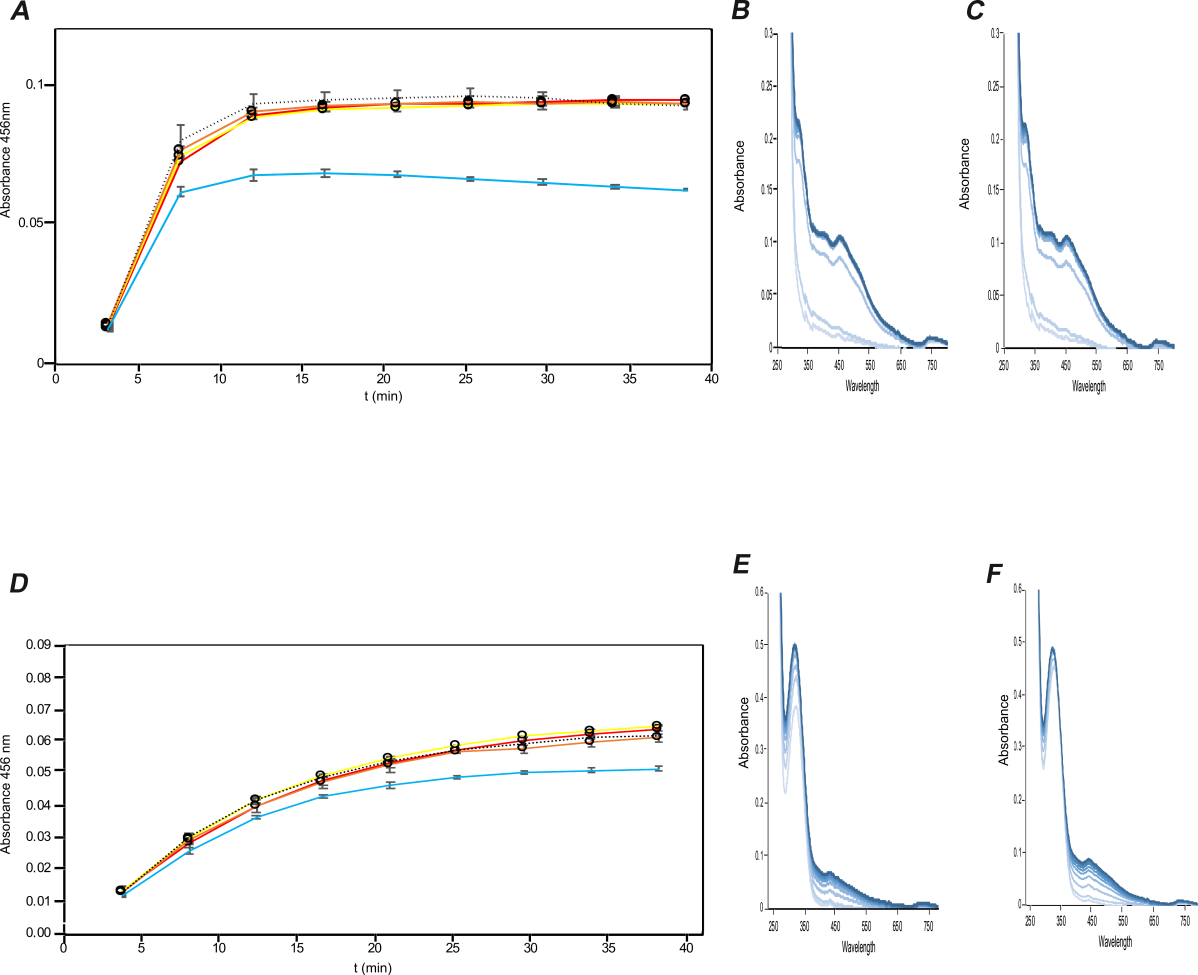
Influence of TusA on Fe-S cluster formation. (**A**) IscU (50 µM) was incubated with Fe^2+^ (25 µM) and TusA at 1, 5, 10, or 50 µM, with either 2 mM DTT(A**–C**) or 1 µM Fdx, 1 µM Fpr, and 100 µM NADPH (**D–F**) in 50 mM Tris/HCl, 100 mM NaCl, pH 8, under anaerobic conditions. After 30 min, IscS (1 µM) was added, and the reaction was started by the addition of 250 µM l-cysteine. Fe-S cluster formation was monitored at 456 nm in 5-min intervals for 40 min. UV-visible absorbance spectra were measured between 250 and 800 nm for samples without TusA and with 50 µM TusA. Time points in UV-vis spectra as color gradient from gray to black. Error bars originate from independent triplicate experiments. Note that the increased intensity of absorbance peaks at 280 and 330 nm for the samples containing Fdx and NADPH results from NADPH absorbance at 340 nm (32). Red line: 1 µM IscS + 50 µM IscU; yellow line: 1 µM IscS + 50 µM IscU + 1 µM TusA; orange line: 1 µM IscS + 50 µM IscU + 5 µM TusA; black-dotted line: 1 µM IscS + 50 µM IscU + 10 µM TusA; blue line: 1 µM IscS + 50 µM IscU + 50 µM TusA. (**B**) and (**E**) Spectra at 5-min time intervals of sample corresponding to the yellow line of the IscS and IscU incubation. (**C**) and (**F**) Spectra at 5-min time intervals of sample corresponding to the blue line of the IscS and IscU and 50 µM TusA incubation. The colors of the spectra are from light to dark blue, with light blue representing the lowest time point and dark blue the highest time point.

Since the formation of an IscS_2_-IscU-TusA heterocomplex has been identified by native mass spectrometry analysis, it was of further interest to determine if TusA influences *in vitro* Fe-S cluster formation, and particularly whether TusA competes with IscU for the persulfide of IscS. In this case, Fe-S cluster formation might be negatively affected by the presence of TusA since not all sulfur would be directed to Fe-S cluster formation but would instead be transferred to TusA for other sulfur transfer pathways. The impact of TusA on Fe-S cluster formation was monitored by using the *in vitro* Fe-S cluster formation assay consisting of IscS, IscU, Fe(NH_4_)_2_(SO_4_)_2_, l-cysteine, and either DTT or Fdx, Fpr, and NADPH as reductant (31). [2Fe-2S] cluster formation was monitored by UV-vis absorbance, see Fig. 5.

Under all conditions tested, TusA did not impact Fe-S cluster formation when present at low concentration, but when at 50 µM (equimolar with IscU), it did significantly impact Fe-S cluster assembly. This was reflected in both the kinetics of cluster formation (as judged through ΔA_456 nm_, Fig. 5A and D) and the overall extent of cluster formation (as judged by the absorbance spectrum, Fig. 5B, C, E, and F) for both experiments, employing DTT (Fig. 5A through C) or FdX/Fpr/NADPH (Fig. 5D through F) as reductants. These results are in agreement with the relative affinities of TusA and IscU for IscS determined by native mass spectrometry: TusA appears to bind IscS only when present at (at least) a concentration around equimolar with IscU, thus competing for IscS persulfide and thereby reducing Fe-S cluster formation.

## DISCUSSION

In the cell, pyridoxal phosphate-containing l-cysteine desulfurases serve as primary sulfur-providing proteins for sulfur-containing biomolecules (33). In *E. coli*, IscS has been shown to be the main sulfur-mobilizing enzyme that provides sulfur for Fe-S clusters, Moco, thiamin, biotin, lipoic acid, and sulfur-containing nucleosides in tRNA (19). To accomplish these multiple roles, IscS interacts with multiple proteins in *E. coli*, including IscU and TusA. The ability of IscS to act as a hub for sulfur transfer might be associated with the length and flexibility of its active-site Cys-containing loop, which enables interactions with proteins bound at different sites on IscS.

High-resolution structural information has revealed (IscS)_2_(IscU)_2_ and (IscS)_2_(TusA)_2_ heterotetramers in which IscU/TusA are bound at distinct but overlapping sites on each IscS monomer (19, 34). A currently unresolved puzzle of IscS function is how sulfur transfer into the various pathways is regulated. Here, we have sought to address the question of how the (IscS)_2_(IscU)_2_ and (IscS)_2_(TusA)_2_ complexes and their activities are affected by the presence of TusA and IscU, respectively.

The affinity of IscU for IscS was previously determined to be *K*_*d*_ ~3 µM, with no evidence of cooperativity between the two IscS subunits (30). IscU occurs in Zn^2+^-bound and apo forms, in addition to the cluster assembly product [2Fe-2S] form; while Zn^2+^ interferes with Fe-S cluster assembly on IscU, it does not significantly affect the affinity of IscU for IscS (30). It is reasonable to assume that [2Fe-2S] IscU would bind to IscS with lower affinity than the cluster-free form because it is not the physiologically relevant substrate. Here, we used similar native mass spectrometry methodology to determine the affinity of TusA for IscS, revealing a similar lack of cooperativity and a *K*_*d*_ ~8 µM. Further studies of an IscS variant lacking an Arg patch (R220/223/225E) that is known to be involved in binding of TusA (19) revealed a significantly lower *K*_*d*_ ~50 µM. On the contrary, binding studies of two Cys variants of TusA (C19S and C56A) revealed unaffected affinities compared to wild-type TusA, consistent with the structure of the IscS-TusA complex in which neither Cys participates in direct interactions with IscS (19).

These affinities suggest that IscU should be able to displace TusA from the (IscS)_2_(TusA)_2_ complex, but higher concentrations of TusA would be needed to displace IscU from the (IscS)_2_(IscU)_2_ complex. This is entirely consistent with the data from gel filtration, where addition of TusA to (IscS)_2_(IscU)_2_ had only a minor effect on the elution profile, but addition of IscU to (IscS)_2_(TusA)_2_ had a very significant effect with essentially full displacement of TusA following addition of a 3:1 excess of IscU over the (IscS)_2_(TusA)_2_ complex.

Displacement of TusA/IscU from complex with IscS could result in the formation of mixed heterocomplexes involving all three proteins, that is, (IscS)_2_(IscU)(TusA). Indeed, this complex was suggested by gel filtration elution profiles and readily detected by native mass spectrometry during titrations of (IscS)_2_(TusA)_2_ with IscU and of (IscS)_2_(IscU)_2_ with TusA. No evidence for any larger complexes was observed, consistent with the previous conclusion that IscU and TusA cannot simultaneously bind the same IscS monomer (19).

Given that TusA activates IscS desulfurase activity threefold (compared to IscS alone) (19, 20) and IscU has little or no effect on IscS activity, we investigated the activities of the mixed heterocomplexes. For cysteine desulfurase activity, addition of excess IscU to the (IscS)_2_(TusA)_2_ complex resulted in a large decrease in activity, consistent with significant displacement of TusA (though not complete displacement suggested by MS that some TusA remained bound). Conversely, addition of excess TusA to (IscS)_2_(IscU)_2_ resulted in only a minor increase in activity relative to (IscS)_2_(IscU)_2_ alone, consistent with little displacement of IscU. For Fe-S cluster formation assays, TusA only began to have a measurable effect when it was present at a concentration similar to that of IscU.

The data are broadly in agreement with previously proposed models in which the concentration of the sulfur acceptor protein is the determining factor for a preferred binding to IscS under particular growth conditions (19–21). While it is known that *iscU*/IscU is regulated by IscR at the transcriptional level, by the small RNA RyhB also at the translational level, and by proteases at the protein level [reviewed in reference (35)], much less is known about the regulation of *tusA*/TusA. Thus, further research will be required to establish the *in vivo* ratios of IscU to TusA and how these vary with growth conditions. However, in addition to relative concentrations of IscU and TusA, relative affinities for IscS are also important, as are the relative desulfurase activities of the IscS complexes. Together, these factors play a key role in regulating sulfur transfer, directing it toward, respectively, Fe-S cluster assembly and Moco synthesis/tRNA modifications. Regulation of sulfur transfer appears to have evolved to favor the assembly of Fe-S clusters that are essential for a broad range of cellular processes, but this can be overcome as levels of IscU and, for example, TusA change according to cellular demand and conditions.

## MATERIALS AND METHODS

### Expression and purification of proteins

IscS (36), TusA (20), and IscU (37) were expressed in BL21(DE3) cells and purified following previously described procedures. Purified IscU were further subjected to (His)6x-tag cleavage by overnight incubation of the purified proteins with 5 mg/mL of thrombin at 4°C and then passed down a Ni^2+^-agarose affinity column to remove the (His)6x-tag. Protein concentrations were determined using the Bradford Reagent Coomassie Plus Protein Assay Reagent (Thermo) with bovine serum albumin as a standard following the manufacturer’s instruction.

### Quantification of l-cysteine desulfurase activity

The activity of IscS or preformed complexes with IscU or TusA was quantified using a methylene blue assay following published procedures (7). For the preformed complexes preparation, purified IscS was incubated with purified IscU or TusA in a ratio of 1:3 for 20 min at 30°C in 50 mM Tris-HCl, 200 mM NaCl, pH 8. The complexes were separated and purified using an equilibrated Superdex 200 pd column (bed volume of 120 mL, Cytiva) connected to an ÄKTA Purifier system (Cytiva). Purified preformed IscS-IscU and IscS-TusA complexes, alone or following the respective addition of TusA or IscU in a 1:3 ratio, were incubated for 10 min at 30°C in the presence of 1 mM DTT and 1 mM l-cysteine. The reaction was stopped by adding acidic 2 mM DMPD and 3 mM FeCl_3_. The produced sulfide was quantified using a standard curve obtained from known concentrations of Na_2_S (0–200 µM). Note that one unit is defined as the amount of enzyme producing 1 µmol of sulfide per minute.

### Protein complex formation studies by analytical gel filtration

For the precomplex formation, 30 µM of purified IscS was incubated with 90 µM of purified IscU or TusA for 20 min at 30°C in 50 mM Tris-HCl, 200 mM NaCl, pH 8.0. The protein mixture was injected onto a Superdex 200 pg column (Cytiva) connected to an ÄKTA purifier system (Cytiva), which has been equilibrated with the same buffer. Proteins were separated at a flow rate of 0.50 mL/min, and the elution profile was recorded at 280 nm. The proteins in the elution fractions were separated using 17% SDS-PAGE. The fractions containing the desired complex were pooled and concentrated.

To test the formation of ternary complexes, IscS-IscU or IscS-TusA complexes were incubated, respectively, with TusA or IscU (1:3 ratio) for 20 min at 30°C, and the same gel filtration procedure was followed. The proteins in the elution fractions were again separated using 17% SDS-PAGE.

### Mass spectrometry

#### Mass spectrometry under non-denaturing conditions

Proteins (IscS, IscU, TusA) were exchanged into 250 mM ammonium acetate, pH 8.0 using PD mini-Trap G25 columns (Cytiva), and the concentration determined using the appropriate calculated molar extinction coefficients (protpram). Samples (200 µL) were prepared immediately prior to use, by dilution, and contained ~8 µM IscS (~4 µM dimeric IscS) together with appropriate ratios of other proteins (IscU, TusA). Samples were infused directly, via syringe pump, into the source of a Bruker micrOTOF-QIII (Bruker Daltonics) or a Waters Synapt XS (Waters Corp.) mass spectrometer, operating in positive-ion mode with a capillary voltage of 3,500 V. Optimization of the experimental conditions for the transmission of dimeric IscS and associated complexes was achieved by increasing the cone voltage to 150 V (135 V is CID on the Bruker instrument). Other parameters were optimized according to Laganowsky et al. (38), behaving in broadly similar ways on Bruker and Waters instruments (39). MS instruments were calibrated with ESI-L low concentration tuning mix (Agilent Tech.) and/or sodium iodide (Waters Corp.). Data were acquired over the *m/z* range 3,000–8,000 for 5-min periods.

For routine LC-MS, a 20 µM aliquot of protein was diluted with an aqueous mixture of 2% (vol/vol) acetonitrile and 0.1% (vol/vol) formic acid, and loaded onto a Proswift RP-1S column (4.6 × 50 mm, Thermo Scientific) attached to an Ultimate 3000 uHPLC system (Dionex, Leeds, UK) with eluant infused into the electrospray ionization source of a Bruker micrOTOF-QIII mass spectrometer (Bruker Daltonics) operating in the positive-ion mode (40).

Processing and analysis of MS experimental data were carried out using Bruker Compass Data analysis v4.1 (Bruker Daltonik) or Waters Mass Lynx v4.2 (Waters Corp.). Neutral mass spectra over the 90–140 kDa range were generated using the maximum entropy deconvolution algorithm of each analysis suite or with UniDec (41). The high *m/z* range in which the large IscS/TusA/IscU complexes and their adducts were detected negatively affected mass resolution for both the Bruker and Waters spectrometers, and, in general, adducts could not be unambiguously identified. Nevertheless, the protein constituents of complexes could be unambiguously determined. Fractional intensities were calculated for each species present during titrations from total ion counts, and data were fitted using the program Dynafit (Biokin), as previously described (12, 42).

## SUPPLEMENTAL MATERIAL

Supplemental material spectrum.00949-24-s0001.docx Fig. S1 to S6.

ASM does not own the copyrights to Supplemental Material that may be linked to, or accessed through, an article. The authors have granted ASM a non-exclusive, world-wide license to publish the Supplemental Material files. Please contact the corresponding author directly for reuse.
