# Supplementary material for: Binding of IscU and TusA to different but competing sites of IscS influences the activity of IscS and directs sulfur to the respective biomolecular synthesis pathway
Source: Microbiol Spectr. 2024 Jul 9;12(8):e00949-24. doi: 10.1128/spectrum.00949-24 (PMC11302665; doi:10.1128/spectrum.00949-24)
Supplement: Supplemental material — Fig. S1 to S6. [file spectrum.00949-24-s0001.docx]

Supporting Information:

**Binding of IscU and TusA to different but competing sites of IscS influences the activity of IscS and directs sulfur to the respective biomolecular synthesis pathway**

Paolo Olivieri^1^, Jason C. Crack^2^ , Angelika Lehmann^1^, Sophie P. Bennett^2^, Nick E. Le Brun^2^*, and Silke Leimkühler^1^*

From the ^1^Institute of Biochemistry and Biology, Department of Molecular Enzymology, University of Potsdam, D-14476 Potsdam, Germany; and the ^2^Centre for Molecular and Structural Biochemistry, School of Chemistry, University of East Anglia, Norwich Research Park, Norwich, NR4 7TJ, UK.

**TABLE OF CONTENTS**

**Supplementary Figures**

Figure S1: Complementation of strain BW25113 with IscS and His_10_-tagged IscS.

Figure S2. ESI-MS investigation of complex formation between IscS and TusA.

Figure S3. LC-ESI-MS analysis of TusA.

Figure S4. ESI-MS investigation of complex formation between R220/223/225E IscS and TusA.

Figure S5. ESI-MS investigation of complex formation between IscS and Cys-to-Ser/Ala variants of TusA

Figure S6. ESI-MS investigation of complex formation between IscS, IscU and TusA.


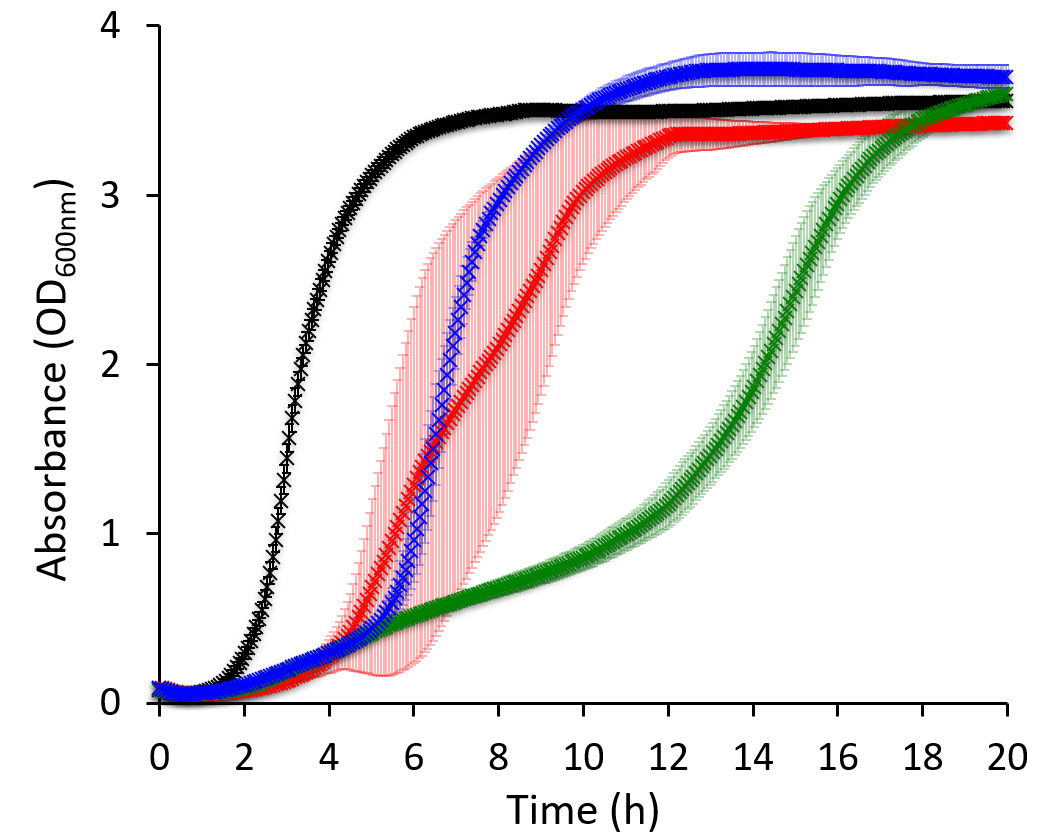


**Figure S1**. **Complementation of strain BW25113 with IscS and His_10_-tagged IscS.** Growth curves of *E. coli* strains BW25113 wild type (black), ∆*iscS* (red), and the ∆*iscS* strain transformed with plasmid pSL209 {Leimkühler, 2001 #1274} expressing untagged IscS (blue), or with plasmid pET11M {Prischi, 2010 #1497} expressing His_10_-tagged IscS (green). Cells were grown in LB medium for 14 h supplemented 100 µM IPTG for the expression of IscS from the indicated plasmids.

**
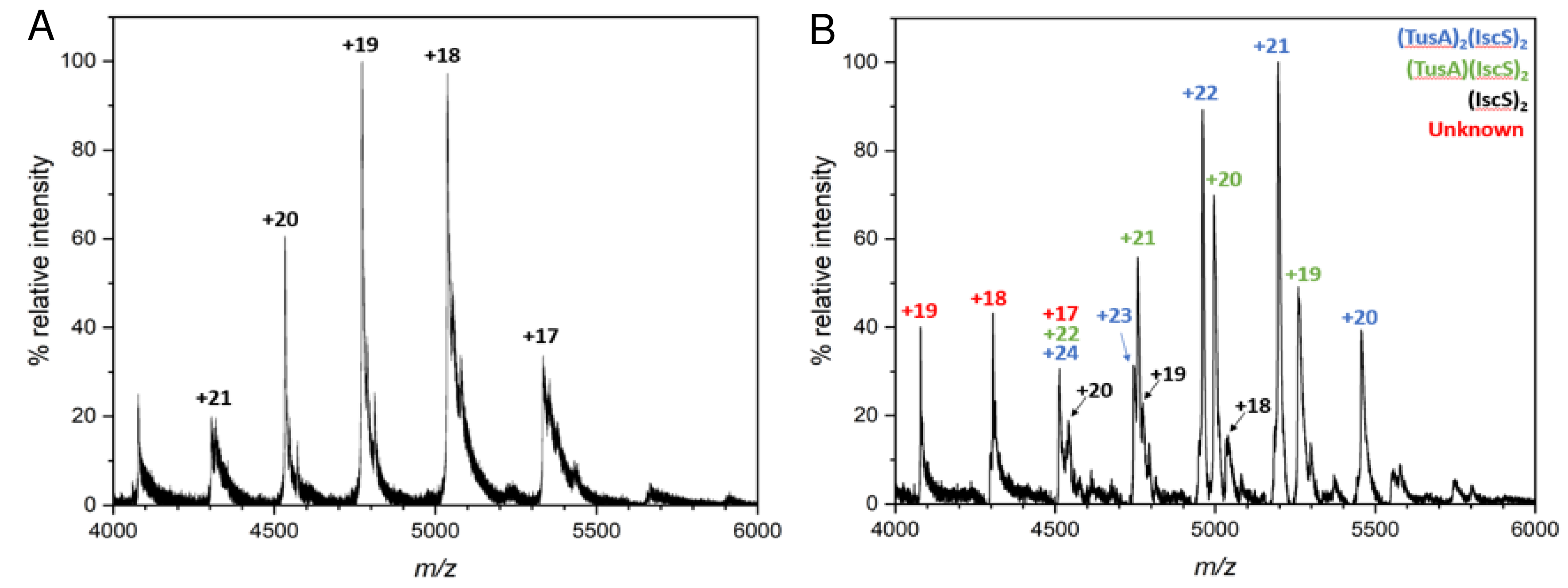
**

**Figure S2. ESI-MS investigation of complex formation between IscS and TusA.** Full *m/z* spectrum for (**A**) IscS (8 µM) and (**B**) IscS following addition of TusA at 2:1 ratio. Charge states corresponding to the various IscS complexes are indicated. Deconvoluted spectra are shown in Figure 3 of the main paper. The unknown species resolved in the presence of TusA appears to be a degraded form of IscS (at 77,487 Da), corresponding to an IscS dimer missing an N-terminal segment, which does not interact with TusA.


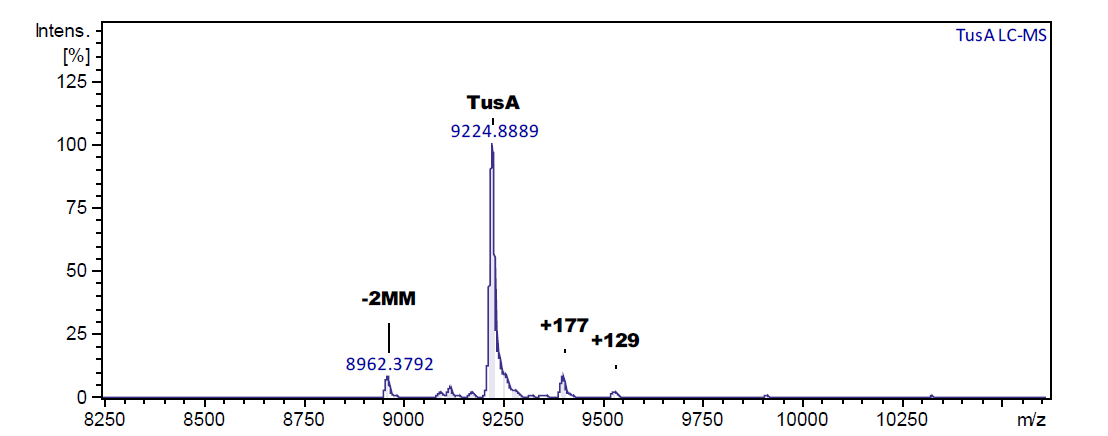


**Figure S3. LC-ESI-MS analysis of TusA.** Deconvoluted spectrum of TusA sample used in this work. The major species correspond to intact TusA, while the lower mass species indicates the presence of a small component of the protein lacking two N-terminal Met residues (-2MM).


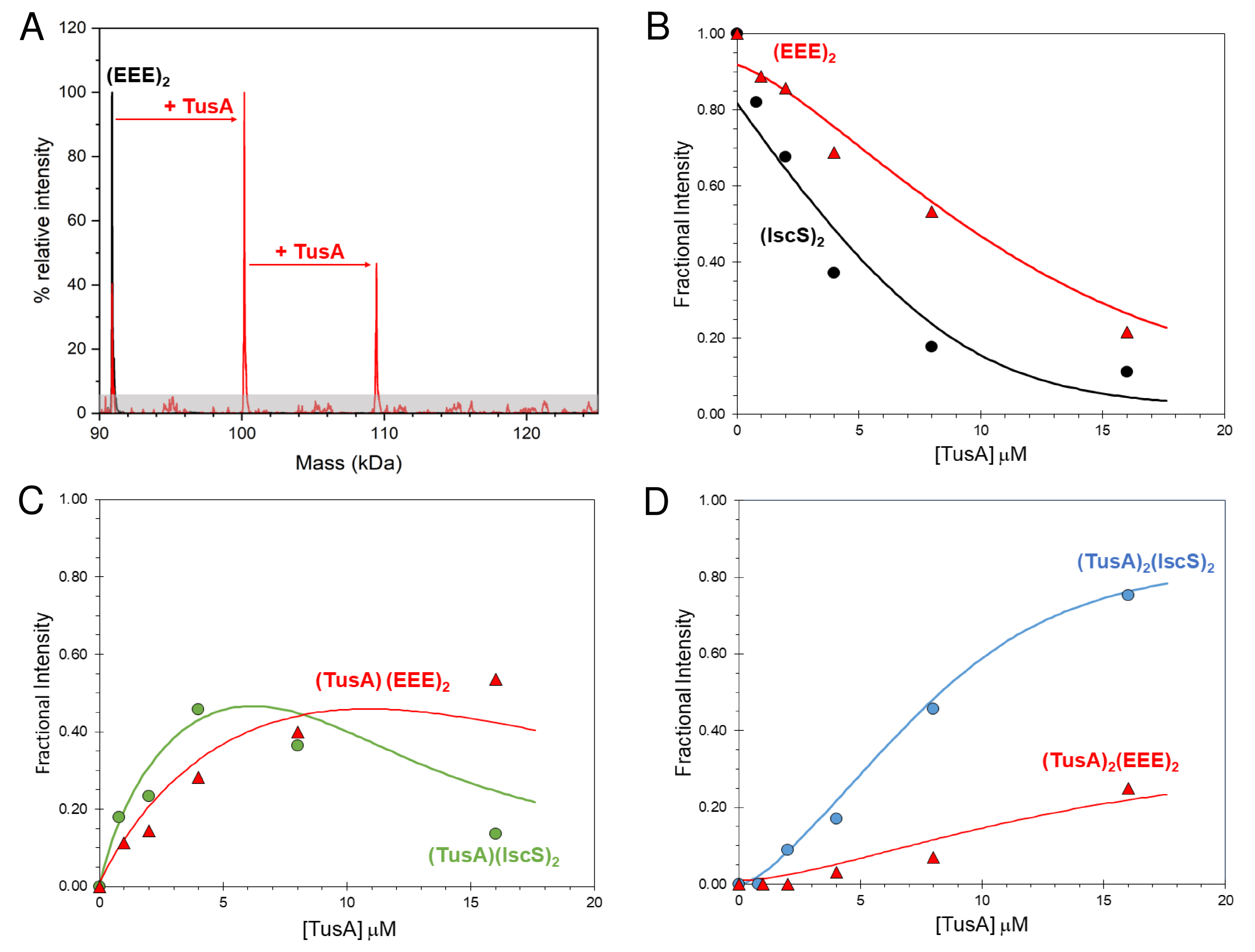


**Figure S4. ESI-MS investigation of complex formation between R220/223/225E** **IscS and TusA**. (**A**) Deconvoluted mass spectrum of IscS over the mass range 90–125 kDa, showing the presence of the IscS dimer (black spectrum). Addition of TusA at a 2:1 excess gave rise to TusA-IscS complexes in which the IscS dimer was bound by one or two TusA protein molecules (red spectrum). We note that the additional mass due to TusA binding suggested a possible zinc adduct of TusA with mass at +64 Da. (**B**) – (**D**) Plots of relative intensity of the various protein complexes, as indicated, as a function of TusA concentration. Data for wild-type IscS binding to TusA are included to aid comparison. Solid lines show fits of the data to a sequential binding model for 1–2 TusA per R220/223/225E IscS dimer. IscS (4 μM dimer) was in 250 mM ammonium acetate, pH 8.

**
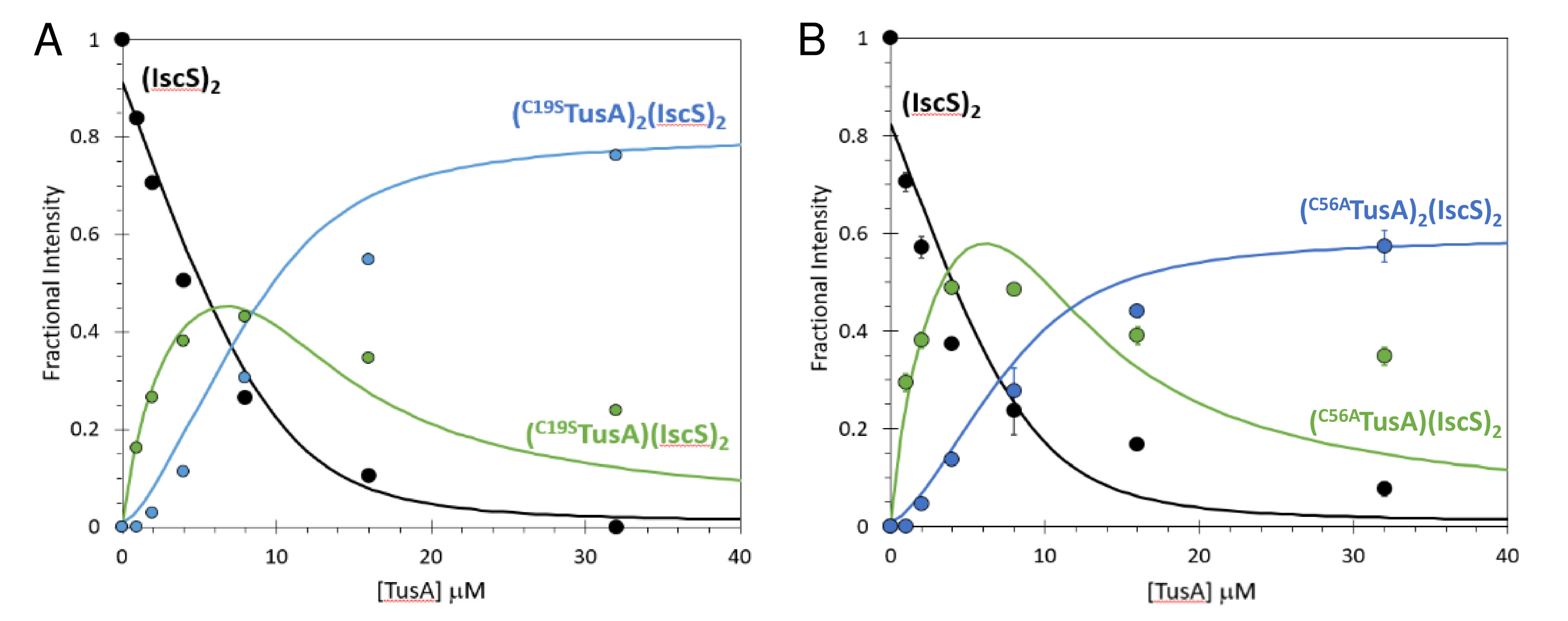
**

**Figure S5. ESI-MS investigation of complex formation between IscS and Cys-to-Ser/Ala variants of TusA**. (**A**) Plots of relative intensity of the various protein complexes, as indicated, as a function of C19S TusA variant concentration. **(B)** as in (A) but data for C56A TusA. We note that the additional mass due to TusA binding suggested a possible zinc adduct of TusA with mass +64 Da. Solid lines show fits of the data to a sequential binding model for 1–2 TusA variants per IscS dimer. IscS (4 μM dimer) was in 250 mM ammonium acetate, pH 8.

**
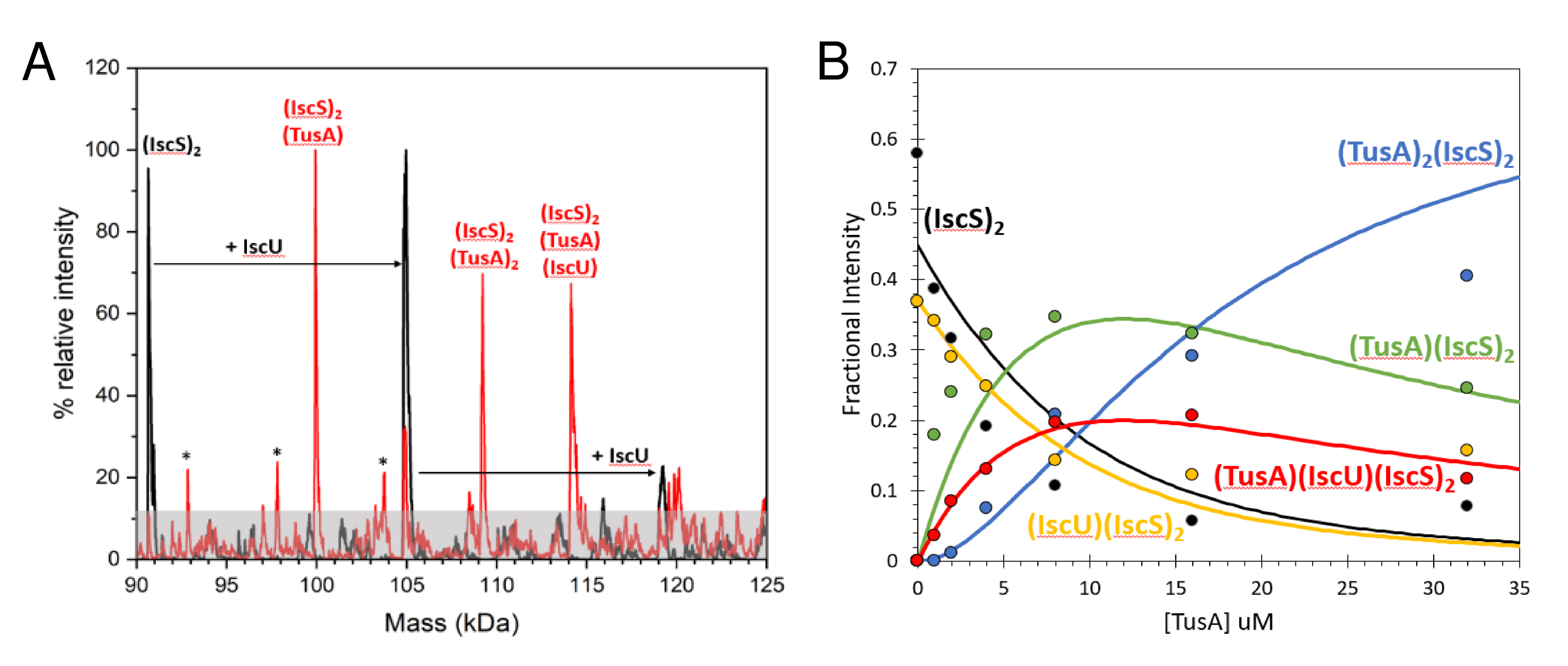
**

**Figure S6. ESI-MS investigation of complex formation between IscS, IscU and TusA**. (**A**) Deconvoluted mass spectrum of IscS complexes over the mass range 90–125 kDa, showing the presence of the IscS dimer and IscU complexes (black spectrum) resulting from the addition of IscU at a 2:1 excess. Subsequent addition of TusA (at 2:1 TusA:IscS/IscU) gave rise to additional complexes, including an (IscS)_2_(TusA)(IscU) species (red spectrum). (**B**) Deconvoluted mass spectral fractional intensities at increasing concentrations of TusA to IscS corresponding to the various protein complexes, as indicated. Solid lines show fits of the data to a competitive binding model for 1–2 IscU or TusA per IscS dimer. Peaks marked with an asterisk in (A) are unknown but could be complexes of degraded forms of IscS with IscU. The mass due to the (IscS)_2_(IscU)_2_ complex appeared to shift by ~500 Da to higher mass following addition of TusA. The reason for this is unknown and the (IscS)_2_(IscU)_2_ species was omitted from the plots of fractional intensity. IscS (4 μM dimer) was in 250 mM ammonium acetate, pH 8.

**REFERENCES**

1. Leimkühler, S., and Rajagopalan, K. V. (2001) A sulfurtransferase is required in the transfer of cysteine sulfur in the in vitro synthesis of molybdopterin from precursor Z in *Escherichia coli*. *J. Biol. Chem.* **276**, 22024-22031

2. Prischi, F., Pastore, C., Carroni, M., Iannuzzi, C., Adinolfi, S., Temussi, P., and Pastore, A. (2010) Of the vulnerability of orphan complex proteins: the case study of the *E. coli* IscU and IscS proteins. *Prot. Express. Purif.* **73**, 161-166
